# A small molecular inhibitor of LRRK1 identified by homology modeling and virtual screening suppresses osteoclast function, but not osteoclast differentiation, *in vitro*

**DOI:** 10.18632/aging.101977

**Published:** 2019-05-21

**Authors:** Mingjue Si, Canjun Zeng, Helen Goodluck, Sandi Shen, Subburaman Mohan, Weirong Xing

**Affiliations:** 1 Musculoskeletal Disease Center, Jerry L. Pettis Memorial VA Medical Center, Loma Linda 92357, CA, USA; 2 Department of Medicine, Loma Linda University, Loma Linda 92350, CA, USA; 3 Department of Orthopedics, The Third Affiliated Hospital of Southern Medical University, Guangzhou, China; * Equal contribution

**Keywords:** LRRK1, bone resorption, bone formation, osteoclast, kinase inhibitor

## Abstract

We used TGFβ activation kinase 1 as a template to build a 3D structure of the human LRRK1 kinase domain (hLRRK1 KD) and performed small molecule docking. One of the chemicals (IN04) that docked into the pocket was chosen for evaluation of biological effects on osteoclasts (OCs) *in vitro*. INO4 at 16 nM completely blocked ATP binding to hLRRK1 KD in an *in vitro* pulldown assay. In differentiation and pit assays, while the number of OCs on bone slices were comparable for OCs treated with IN04 and DMSO, IN04 treatment of OCs significantly impaired their ability to resorb bone. The area of pits on bone slices was reduced by 43% at 5 μM and 83% at 10 μM as compared to DMSO. Individual pits appeared smaller and shallower. F-actin staining revealed that DMSO-treated OCs displayed clear actin rings, and F-actin forms a peripheral sealing zone. By contrast, IN04-treated OCs showed disarranged F-actin in the cytoplasm, and F-actin failed to form a sealing zone on bone slices. IN04 treatment had no effects on OC-derived coupling factor production nor on osteoblast nodule formation. Our data indicate IN04 is a potent inhibitor of LRRK1, suppressing OC function with no effect on OC formation.

## INTRODUCTION

Osteoporosis is an aging-related major health problem in the United States. There are two major known causes of osteoporosis: low peak bone mineral density (BMD), typically achieved around the age of 30, and a high rate of bone loss that occurs particularly after menopause and during the natural process of aging. Bone loss occurs with age in part because the rate of bone resorption surpasses the rate of bone formation. Effective inhibitors of bone resorption such as bisphosphonates have widely been used in the clinic to treat high-turnover bone diseases. However, treatment with bisphosphonates results in suppression of both bone resorption and bone formation and blunts the anabolic actions of PTH [1]. Because bisphosphonates are incorporated within the bone matrix with high affinity, long term treatment with these drugs may impair fracture healing, cause jaw osteonecrosis, and increase the risk for atypical fractures of the femur [2–7]. Although a cathepsin K inhibitor had shown promise in large animal and human clinical studies, treatment with the cathepsin K inhibitor caused off-target effects and increased the risk of stroke [8]. Treatment of postmenopausal women with an anti-RANKL (receptor activator of nuclear factor-κB ligand) monoclonal antibody (denosumab) was effective, however the bone turnover rebounds after 2 years’ discontinuation of denosumab [9]. Therefore, novel anti-resorptive molecules that avoid anti-anabolic actions are needed as therapeutics to increase bone mass and reduce fracture risk.

Leucine rich repeat kinase 1 (LRRK1) belongs to the ROCO protein family and contains four ankyrin repeats (ANK), seven leucine-rich repeats, a GTPase-like domain of ras of complex proteins (Roc), a C-terminal of Roc domain (COR), and a serine/threonine kinase domain that is regulated by GTP binding to the Roc domain [10]. A point mutation within the Roc domain that abolishes GTP/GDP binding resulted in inaction of LRRK1 kinase *in vitro* [11, 12]. In our previous studies, we have demonstrated that mice with disruption of the *Lrrk1* gene displayed severe vertebral and long bone osteopetrosis resulting from the dysfunction of mature osteoclasts (OCs). OCs lacking LRRK1 failed to form peripheral sealing zones on bone slices due to defects in RANKL-induced cytoskeletal rearrangement. In contrast to bisphosphonate-treated monocytes, precursors derived from *Lrrk1* KO mice differentiate normally into mature OCs, and these OCs fail to resorb bone because of reduced OC activity. While bone resorption in KO mice is reduced dramatically, bone formation is not significantly affected [1]. *Lrrk1* KO mice have normal teeth, are healthy through one year of age, and respond to anabolic PTH treatment, but they are resistant to ovariectomy-induced bone loss [1]. More recently, an autosomal recessive mutation of *Lrrk1* has been identified in a human patient [13]. A partial DNA deletion in the *Lrrk1* gene caused a frame-shift mutation, resulting in the disruption of the 7^th^ WD-40 repeats and addition of a 66 amino acid sequence to the C-terminus of the LRRK1 protein. The mutation caused loss of LRRK1 function in OCs. The clinical features of the patient were very similar to the skeletal phenotypes observed in the *Lrrk1* KO mice. The patient with a loss of function mutation had an osteosclerotic metaphyseal dysplasia, a distinctive form of osteopetrosis characterized by severe osteosclerosis confined to the metaphysis of the long and short tubular bones due to OC dysfunction [1, 13]. These studies strongly suggest that LRRK1 plays a critical role in regulating OC function and peak bone mass. Therefore, LRRK1 is a novel drug target for alternative anti-resorptive drugs to treat osteoporosis and osteoporotic fractures.

The 3D structures of the ROCO4 superfamily including LRRK1 and LRRK2 can be used as a receptor for structure-based drug screening [14]. In previous studies, the 3D structure of the ROC domain dimer from LRRK2 was resolved and was used for a combination of computer-aided drug design for screening small molecule competitors against the GTP pocket for treatment of Parkinson disease [15, 16]. Little is known about the structure of the LRRK1 KD. In this study, we performed structure homology modeling and virtual screening [17], and we tested the anti-resorptive function of a candidate LRRK1 inhibitor *in vitro*.

## RESULTS

### IN04 docks to the active pocket of the LRRK1 KD and inhibits ATP binding to the LRRK1 KD

**Figure 1:**
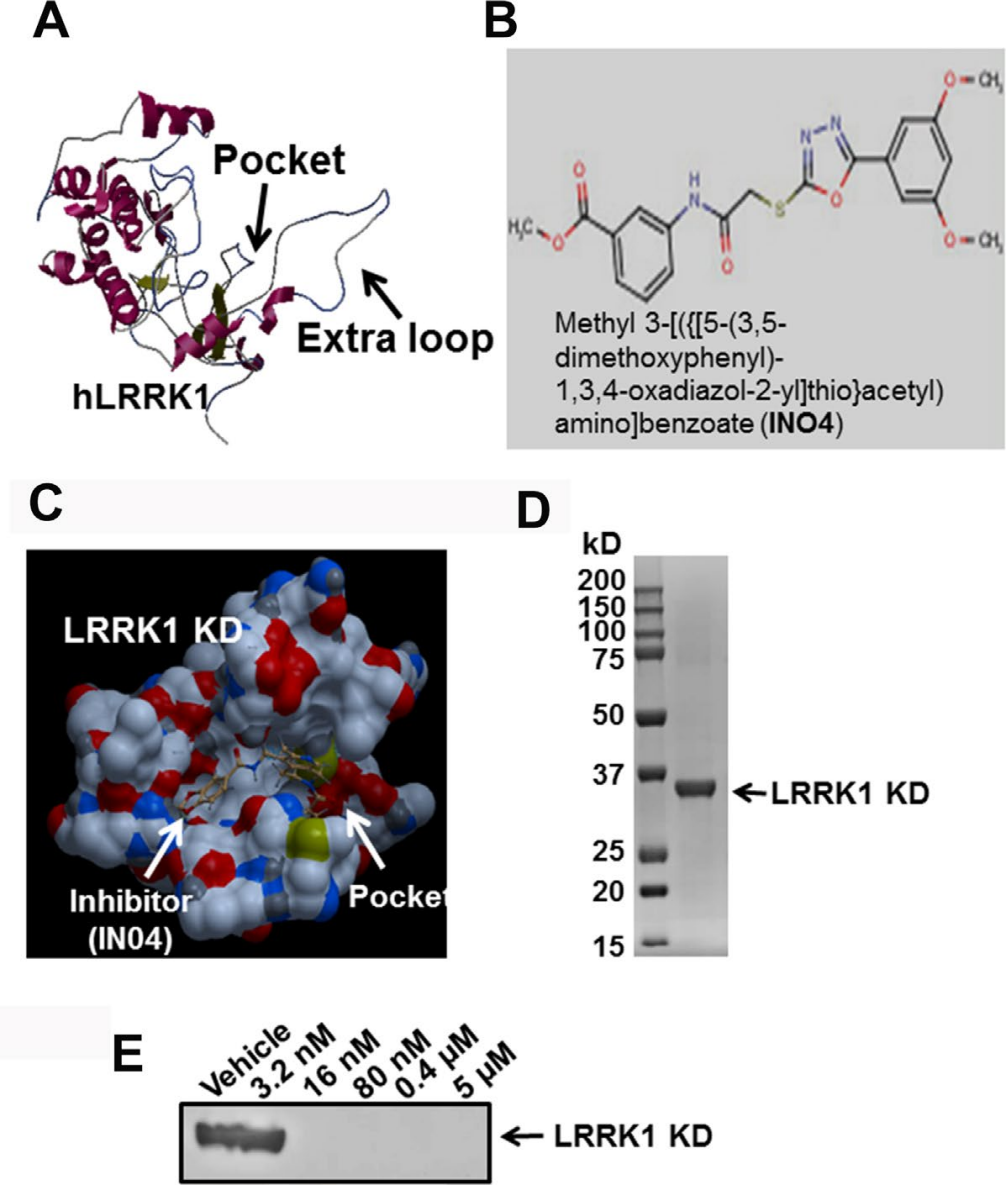
**Small molecular inhibitor IN04 binds to the predicted active pocket of hLRRK1 kinase domain.** (**A**) Predicted structure of hLRRK1 kinase domain with an active pocket. (**B**) A molecular structure of the potential LRRK1 inhibitor IN04. (**C**) A potential small molecular weight inhibitor docks to the active pocket of hLRRK1 KD. (**D**) Purified 34 kD recombinant protein of hLRRK1 expressed in *E. coli* stained with Coomassie blue. (**E**) IN04 inhibits ATP binding to the LRRK1 KD, measured by an *in vitro* pulldown assay.

Homology-based protein modeling of the hLRRK1 KD indicated that the LRRK1 KD contains an extra loop in the activation site compared with the hLRRK2 KD [18], and it has a narrower active pocket for ligand binding (Figure 1A). A total of approximately 5,000 KINACore and 11,000 KINASet compounds were screened. By using a computer program, four promising inhibitors (Cambridge, #20040537, #3148851, #21337060, and #7989904) were identified with high scores (data not shown). Among them, IN04 (#7989904) not only docked to the active pocket of the hLRRK1 KD but also inhibited OC function without suppressing OC formation in OC differentiation and pit assays (Figure 1B, 1C). Two of these compounds inhibited OC formation (#3148851 and #21337060) in the OC differentiation assay, and one of these compounds (#20040537) did not suppress OC function either in pit formation assays, although it did not inhibit OC formation (data not shown). To test if IN04 docking to the active pocket inhibits ATP binding to the LRRK1 KD, we first purified recombinant protein from *E. coli* and used the protein for an *in vitro* pulldown assay. We found that INO4 at 16 nM completely blocked ATP binding to the LRRK1 KD (Figure 1D, 1E).

### IN04 treatment inhibits OC bone resorption function with no effect on OC formation

**Figure 2:**
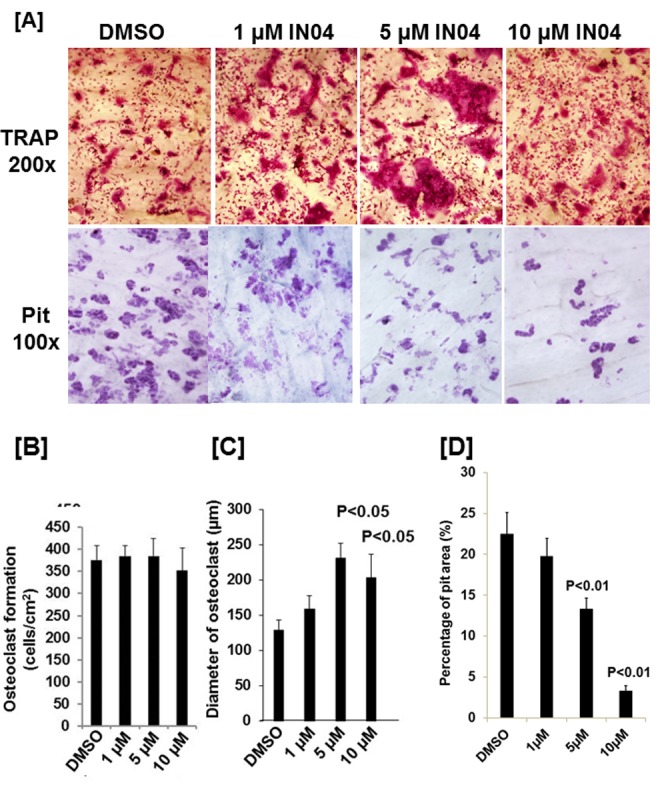
**IN04 treatment inhibits osteoclast bone resorption function with no effect on osteoclast formation.** Osteoclast precursors derived from C57BL/6J mice were seeded on bone slices (0.4 x 0.8 cm) and differentiated in the presence of DMSO or INO4 for 6–9 days. Cells were stained for TRAP (Tartrate-resistant acid phosphatase), and bone slices were stained with hematoxylin for bone resorption pits. (**A**) Representative images of TRAP-positive osteoclasts (upper panel) and resorption pits (lower panel) on bone slices. (**B**) Quantitative data of osteoclast numbers on bone slices. (**C**) Quantitative data of osteoclast size on bone slices. [29]. Quantitative data of pit formation on bone slices. The results are presented as percentage of pit area relative to the bone slice area. Data are presented as mean ± SEM. *P* < 0.05 or *P* < 0.01 indicates statistical significance (N=4).

**Figure 3:**
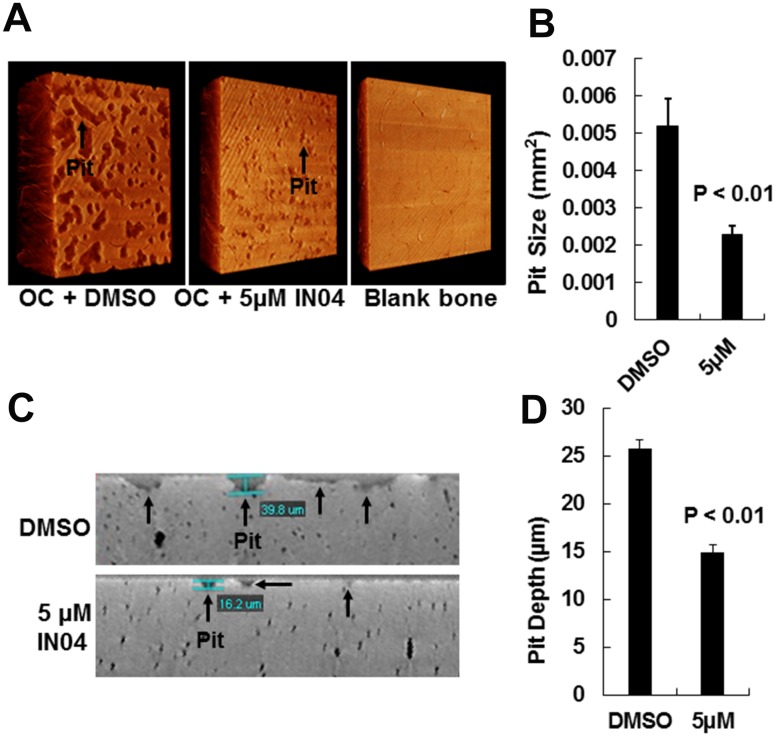
**IN04 treatment impairs osteoclast function.** (**A**) Representative images of bone resorption pits on bone slices scanned by nano-CT. Bone slices described in Figure 5, and a blank bone slice (1.5x1.5 mm) was scanned by nano-CT is shown. (**B**) Quantitative data of pit size in area on bone slices. (**C**) Representative images of vertical section of bone resorption pits on bone slices. (**D**) Quantitative data of pit depth on bone slices, measured by nano-CT. Data are presented as mean ± SEM. *P* < 0.05 or *P* < 0.01 indicates statistical significance (N=3).

**Figure 4:**
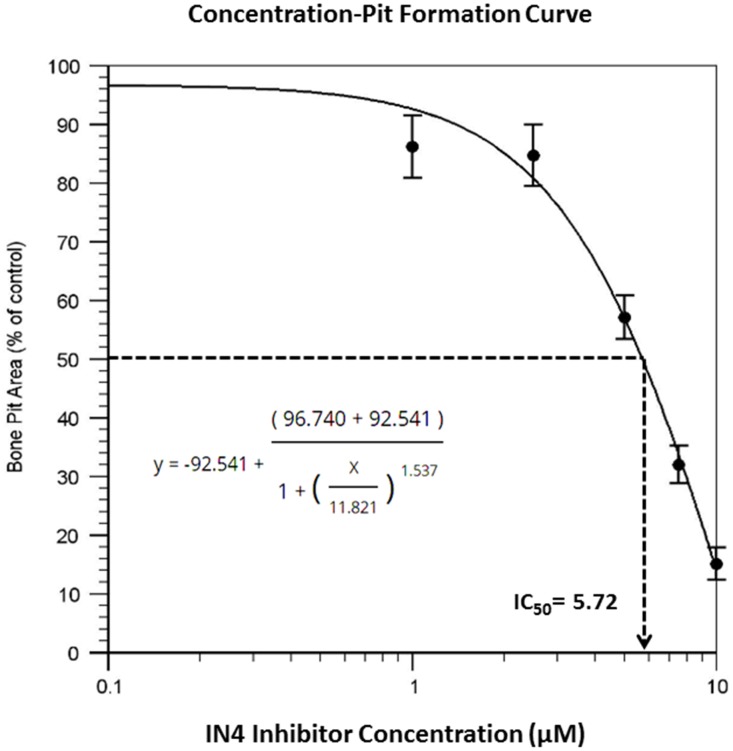
**Dose response curve of resorptive pit formation.** Osteoclast precursors derived from C57BL/6J mice were seeded on bone slices and differentiated in the presence of DMSO or the indicated concentrations of INO4 for 6–9 days followed by hematoxylin staining for bone resorption pits. The results are presented as percentage of pit formation relative to the DMSO-treated control. Results are averages of duplicates with comparable results obtained in another independent experiment (N=4). The IC_50_ value was derived from the graph.

To test the effect of IN04, we performed OC formation and bone resorption pit assays *in vitro*. CD11b-positive monocytes isolated from the mouse spleen were seeded on bone slices and treated with 20 ng/ml M-CSF and 30 ng/ml RANKL in the presence of candidate inhibitor or vehicle DMSO to form multinuclear cells. The mature OCs on the bone surface were counted. Bone resorptive pits were stained with hematoxylin after removing the cells from the bone surface. We observed that INO4 treatment had no effect on OC formation but significantly inhibited bone resorption in a dose-dependent manner (Figure 2A). While the number of mature TRAP-positive OCs on bone slices were comparable for the IN04 and DMSO treatments, IN04 treatment of OCs increased OC fusion, and significantly impaired their ability to resorb bone without causing toxicity or cell death. Dysfunctional OCs treated with IN04 kept fusing and became larger than the DMSO-treated control cells (Figure 2C). The average area of IN04 treated OCs on bone slices at 5 μM and 10 μM was 6 and 3 times bigger than the DMSO treated cells, respectively. The area of resorption pits was reduced by 43% in the cultures at 5 μM IN04 and 83% at 10 μM IN04 as compared to DMSO vehicle-treated control cells (Figure 2B, 2D). In addition, individual resorption pits appeared smaller and shallower in IN04 treated cultures as measured by nano-CT (Figure 3A, 3C). Pit size on bone slices was reduced by 54%, and pit depth was decreased by 42% (Figure 3B, 3D). The average pit size was 2.4 x10^-3^ mm^2^ in the IN04-treated cultures vs. 5.2 x10^-3^ mm^2^ in the vehicle controls. Pit depth on bone slices was 15 μM in the IN04-treated vs. 26 μM in the controls. By resorptive pit formation assays, we have estimated a 50% inhibition of resorptive function (IC50) at an IN04 concentration of 5.72 ± 0.86 μM *in vitro* as shown in Figure 4. In addition, we tested 3D analogs that are structurally like IN04 from the Chembridge small molecular libraries (#7929558, #7976361, #7386352, #7966678, #7962797, and #7927030), and none of them could inhibit bone resorption *in vitro* assessed by pit formation assays (data not shown).

### IN04 treatment of OCs impairs peripheral sealing zone on bone slices

**Figure 5:**
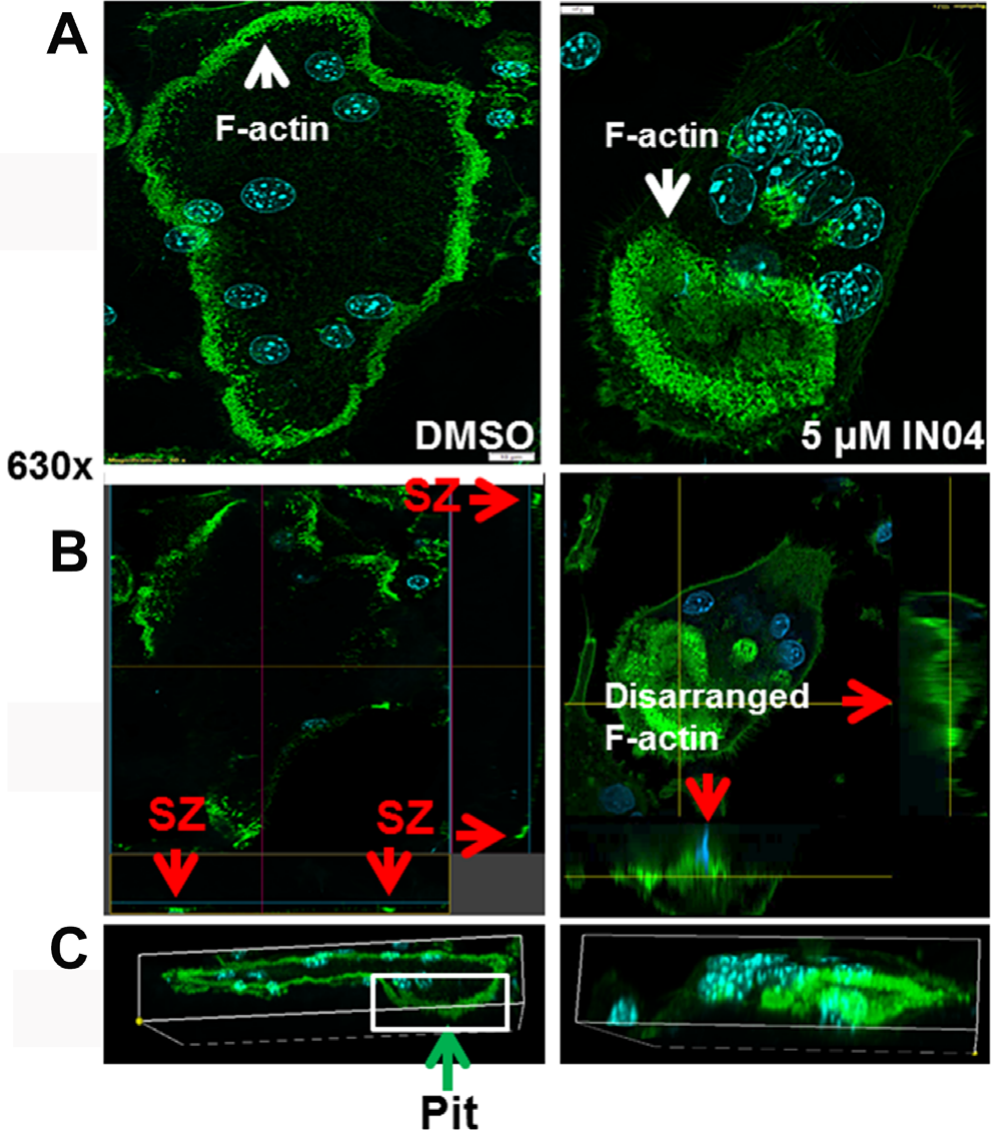
**IN04 treatment disrupts the cytoskeleton arrangement and F-actin ring formation of osteoclasts.** Osteoclast precursors derived from C57BL/6J mice were seeded on bone slices and differentiated in the presence of DMSO or INO4 for 6–9 days. Mature osteoclasts were stained with Alexa Fluor 488-conjugated phalloidin and DAPI, and the actin ring formation and sealing zone were visualized by confocal microscopy. (**A**) Representative images of IN04- and DMSO-treated osteoclasts. (**B**) Horizontal cross sections of selected osteoclasts. Two lines in the middle of the cell represent positions of horizontal and vertical cuts, respectively. Rectangles are orthogonal views of X- and Y-sections, respectively. Arrows in red indicate sealing zones (SZ). (**C**) 3D rendering of a lateral view. A DMSO-treated osteoclast was invaded in a resorptive pit indicated by an arrow in green while an IN04-treated cell was on the surface of the bone slice.

As shown in Figure 5, almost all vehicle-treated mature OCs displayed a typical rounded appearance with clear actin ring formation (Figure 5A, left panel). Horizontal cross-section images of multinucleated cells revealed clustered F-actin formed a peripheral sealing zone (SZ) in DMSO-treated OCs from both views when cultured on bone slices (fluorescent dots indicated by arrows in Figure 5B, left panel). These mature OCs were associated with resorptive pits as shown in Figure 5C, left panel. By contrast, more than 90% of IN04-treated multinucleated cells showed disarranged F-actin in the cytoplasm with weak peripheral F-actin rings. Despite contact with the bone surface, IN04-treated OCs failed to form a typical sealing zone, and the cells were not associated with resorptive pits (Figure 5B, 5C, right panel).

### IN04 treatment does not influence OC differentiation and coupling factor expression

**Figure 6:**
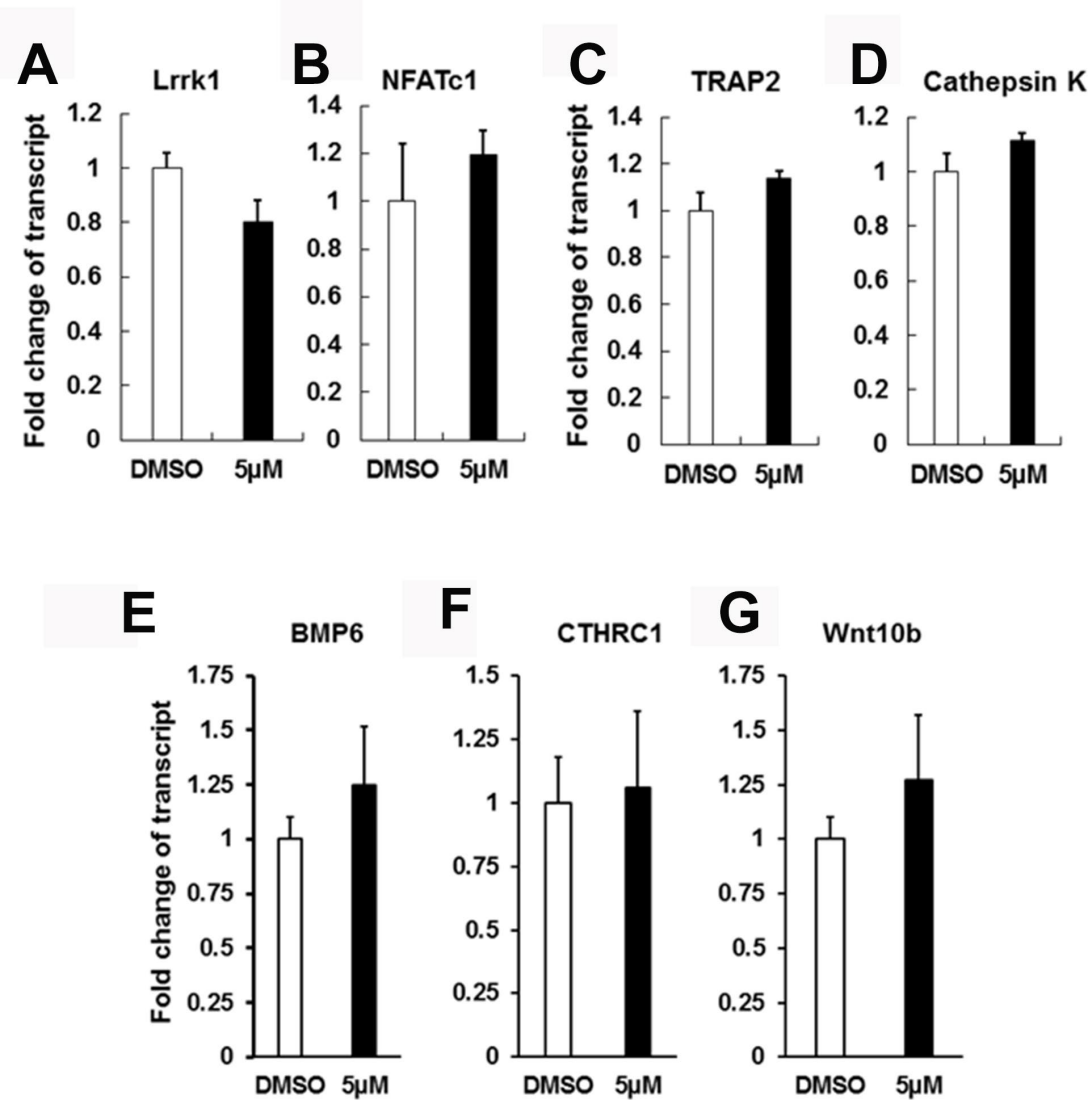
**IN04 treatment does not influence osteoclast differentiation and coupling factor expression.** Osteoclast precursors derived from C57BL/6J mice were cultured in 6-well plates and differentiated in the presence of DMSO or INO4 for 6–9 days followed by RNA extraction and real-time RT-PCR. Expression levels of endogenous Lrrk1 and osteoclast differentiation markers NFATc1, Acp5, and Cathepsin K, respectively (N=6). Expression levels of osteoclast coupling factors BMP6 (bone morphometric protein 6), CTHRC1 (collagen triple helix repeat containing 1), and Wnt10b (wingless-type MMTV integration site family, member 10B), respectively (N=6). Data are presented as mean ± SEM. *P* < 0.05 or *P* < 0.01 indicates statistical significance.

To test if IN04 treatment of mature OCs alters expression of OC differentiation marker genes and OC-derived coupling factors, including bone morphometric protein 6 (BMP6), collagen triple helix repeat containing 1 (CTHRC1), and wingless-type MMTV integration site family member 10B (Wnt10b), we cultured CD11b-positive precursors derived from mouse spleens and differentiated the cells on bone slices in the presence of M-CSF and RANKL together with DMSO or IN04 (5 μM) for 6 days. The mature multinuclear cells were harvested for RNA extraction and real-time RT-PCR. We found that IN04 inhibition mature OC function did not compromise the expression of endogenous Lrrk1 or the OC differentiation marker genes: nuclear factor of activated T cells cytoplasmic, calcineurin dependent 1 (NFATc1), TRAP, and cathepsin K in mature OCs (Figure 6A–6D). The expression levels of known OC coupling factors such as BMP6, CTHTC1, and Wnt10b were unchanged in IN04-treated OCs as compared to vehicle-treated cells (Figure 6E–6G).

### IN04 treatment has no effect on bone marrow stromal cell-derived nodule formation *in vitro*

**Figure 7:**
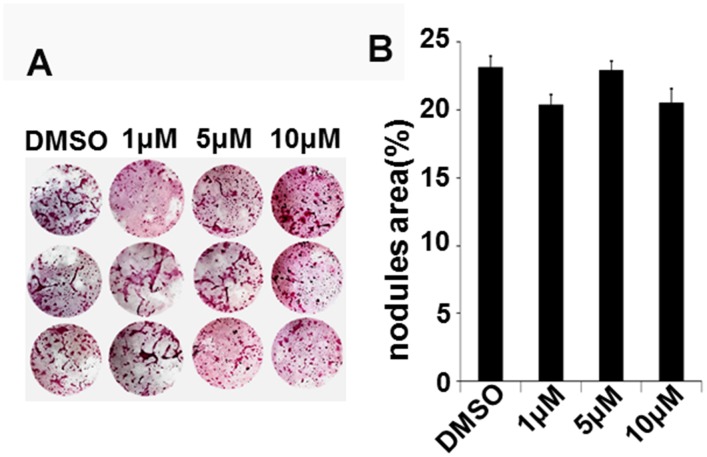
**IN04 treatment has no effect on nodule formation *in vitro***. Bone marrow stromal cells were treated with a mineralization medium containing 10 mM β-glycerophosphate, 50 μg/ml ascorbic acid, and 10% FBS in the presence of DMSO or INO4 for 24 days and stained with alizarin red. (**A**) Images of nodule formation. (**B**) Quantitative mineralization area measured by the OsteoMeasure system. Data are presented as mean ± SEM, N=3.

To evaluate whether IN04 has effects on osteoblast differentiation, we cultured bone marrow stromal cells and differentiated them to form nodules *in vitro*. We found that the amount of mineralized nodule formation was comparable in IN04-treated bone marrow stromal cells as compared to DMSO-treated cells after 24 days of culture in a mineralization medium (Figure 7A, 7B).

## DISCUSSION

The ideal agents for management and treatment of osteoporosis should have both OC inhibitory and osteoblast stimulatory functions. However, such drugs are currently not available. At present, most available drugs function to limit bone resorption, either directly or indirectly targeting OCs. These antiresorptive agents include bisphosphonate family members (e.g., alendronate, Fosamax, Reclast, or Bovina), the selective estrogen receptor modulator raloxifene, and a fully humanized monoclonal antibody specific to RANKL. Contrasting with the anti-resorptive drugs are the anabolic drug agents that stimulate skeletal bone formation. These drugs consist of full-length parathyroid hormone (PTH) 1–84, PTH (1–34), and the PTH related peptide analog abaloparatide. Given the current limited options for regulation of bone anabolism and the side effects of current anti-resorptive drugs due to suppression of OC differentiation and OC–osteoblast coupling, it is important to develop additional alternatives that can trigger bone formation or novel anti-resorptive agents that suppress bone resorption without inhibiting mature OC-coupled bone formation. In this study, we chose LRRK1 as a drug target for anti-resorptive drug screening because our previous studies have demonstrated that a lack of LRRK1 in mice resulted in increased bone mass and prevented OVX-induced bone loss in aging mice. *Lrrk1* KO mice were healthy and responded normally to anabolic PTH treatment. In contrast to bisphosphonate-treated monocytes, precursors derived from *Lrrk1* KO mice differentiated normally into mature multinuclear cells that failed to resorb bone [1]. Our previous studies suggest different mechanisms for bisphosphonate and LRRK1 actions on bone cells. We predicted that inhibition of LRRK1 action by small molecular weight inhibitors in OCs would inhibit OC function with no effects on OC–osteoblast coupling. As expected, we found that IN04 treatment of OCs significantly impaired their ability to resorb bone by blocking ATP binding to the active pocket of LRRK1 KD. Individual pits appeared smaller and shallower while OC formation on the bone slices was normal. IN04-treated OCs exhibited disorganized F-actin in the cytoplasm, and F-actin failed to assemble a sealing ring on bone slices. The phenotypic features of mature OCs treated with IN04 were very similar to the dysfunctional OCs derived from *Lrrk1* KO mice that were reported previously [1]. Our results indicate that the inhibitor IN04 may suppress OC activity via inactivation of LRRK1 function in mature OCs.

Mature OCs are known to send OC-derived signals to promote osteoblast precursor recruitment and differentiation and as such stimulate bone formation [19, 20]. There are 3 main classes of OC-derived coupling factors: (1) OC-mediated release of stored growth factors from bone matrix during bone resorption, (2) mature OC-synthesized and secreted factors, and (3) OC membrane proteins. Among them, OC-secreted Wnt10b and CTHRC1, a glycoprotein associated with Wnt family signaling, are two important enhancers of bone formation [21–23]. Interestingly, expression of OC-derived Wnt10b is induced by TGFβ, and the ability of OCs to stimulate bone formation in response to TGFβ could be blocked by the Wnt10b inhibitor DKK-1 [20]. Mice with disruption of Wnt10b exhibited aging-dependent loss of bone mass and progressive reduction of mesenchymal progenitor cells [24]. CTHRC1 is also secreted by mature OCs in the active resorbing bone, and its expression is highly induced by BMP2, hydroxyapatite, and RANKL but blunted by aging and alendronate treatment [23, 25]. *Cthrc1*-null mice displayed low bone mass because of decreased bone formation and osteoblast number, whereas *Cthrc1* transgenic mice showed high bone mass due to increased osteoblast number. Both *Cthrc1*-null and transgenic mice have normal OC number [23, 25]. Taken together, these studies indicate that CTHRC1, Wnt10b, and BMP6 could be involved in the cross-talk between OCs and osteoblasts during bone remodeling. Thus, if our hypothesis that IN04 inhibits OC function without influencing bone formation is correct, we should observe no changes in the expression of OC-derived coupling factors. As predicted, we found that IN04 treatment had no effect on expression of CTHRC1, BMP6, and Wnt10b in mature OCs *in vitro*. Additionally, IN04 treatment did not affect osteoblast functions since the amount of mineralized bone formed by bone marrow stromal cells *in vitro* was not affected by IN04 treatment. Our data indicate IN04 is a potent inhibitor of LRRK1 that suppress OC function with no effect on OC formation or osteoblast functions *in vitro*. However, whether IN04 has the same inhibitory effect on OC function and OC-osteoblast coupling *in vivo* needs to be examined in future studies. Based on the *in vitro* studies, we cannot exclude the possibility that IN04 decreases release of bone-matrix derived growth factors and, thereby, bone formation *in vivo* due to impaired bone resorption.

Direct high throughput screening of small molecular libraries for LRRK1 inhibitors by testing inhibitory phosphorylation or autophosphorylation via a LRRK1 kinase assay is not feasible because the key direct biological substance(s) and autophosphorylation site(s) have not been identified yet. In this study, we first screened the compounds designed to target the ATP-binding site against LRRK1 kinase by computer program-based virtual screening, and we subsequently confirmed the efficacy by using an *in vitro* ATP-site competition binding assay and cell-based functional testing. The screening strategy enabled rapid identification of candidate compounds that selectively inhibited OC function but not OC differentiation. The identified candidate inhibitor of LRRK1, IN04, was not able to dock into the active pocket of LRRK2 KD and was structurally distinct from LRRK2-IN01 inhibitor that suppresses LRRK2 but not LRRK1 kinase activity by blocking ATP binding to the LRRK2 kinase domain [26]. The PubChem (CID 1259183) database showed that it was inactive to MITF (melanogenesis associated transcription factor) and STK33 (serine/threonine kinase 33). The 3D analogs of IN04 failed to inhibit OC function. Our studies indicate that IN04 is relatively selective inhibitor of LRRK1 kinase.

Since the 3D structure of Lrrk1 is not available, we used TAK1 as a template to build 3D structures of hLRRK1 KD for drug screening. Thus, it is possible that IN04 may inhibit enzymatic activity of TAK1 or other MAPKKK family members. Although we minimized off-target effects by eliminating the compounds that also interact with other known kinases (e.g., p38 MAP kinase, PKC, GSK, c-Src, and B-RAF kinase) and focused on compounds that suppress OC activity by disrupting cytoskeleton rearrangement, but not through affecting OC differentiation, further characterization of the kinase selectivity of IN04 are still needed with experimental high-throughput approaches such as kinase binding assays by the KINOMEscan^TM^ methodology and activity-based enzymatic assays by the KiNativ^TM^ technology [27, 28]. *In vivo* testing in ovariectomized osteoporotic mice or inflammatory bone mouse models with high bone turnover is also needed to confirm that IN04 inhibits bone resorption with no effect on bone formation and to determine whether IN04 has better pharmacologic efficacy than bisphosphonates in treating osteoporosis. It will be our future direction to conjugate IN04 inhibitor with penetrating oligopeptide (DSS)_6_ to target active OCs on bone surface. Lastly, a crystallographic structure of LRRK1 needs to be resolved for refinement of potential LRRK1 inhibitors and for high throughput screening of more specific and selective inhibitors.

## MATERIALS AND METHODS

### Mice

Five-week-old C57BL/6J mice were purchased from the Jackson Laboratory (Bar Harbor, ME). Mice were housed at the VA Loma Linda Healthcare System (VALLHCS) under standard approved laboratory conditions. All procedures were performed with approval of the Institutional Animal Care and Use Committee of VALLHCS.

### Recombinant proteins, antibodies, and plasmids

Recombinant macrophage colony stimulating factor (M-CSF) and receptor activator of nuclear factor-κB ligand (RANKL) and anti-6xHis antibody were from R&D Systems (Minneapolis, MN). Ni resin-based columns were obtained from Bio-Rad (Hercules, CA). Plasmid of pUC57-hLrrk1 KD encoding human LRRK1 kinase domain (hLRRK1 KD) from amino acids 1240–1530 was synthesized by GenScript (Piscataway, NJ) and sub-cloned into the pET28a plasmid with 6xHis tag at the N-terminus for recombinant protein expression in *E. coli*. ATP Affpur kit III was purchased from Jena Bioscience (Jena, Germany). The chemical inhibitor methyl 3-[({[5-(3,5-dimethoxyphenyl)-1,3,4-oxadiazol-2-yl] thio} acetyl) amino] benzoate (IN04) was from Cambridge (San Diego, CA).

### hLRRK1 KD expression and purification

The recombinant hLRRK1 KD protein was expressed in Rosetta competent cells in LB broth containing 2 mM MgSO_4_ and induced by a 12-hour treatment of 4 mM *IPTG* at 25°C. The cells were lysed in a lysis buffer containing 25 mM Tris-HCl (pH 8.0), 300 mM NaCl, and 10% N-Lauroylsarcosine sodium (Sarkosyl) by sonication. The lysate was diluted 10 times with a buffer containing 25 mM Tris-HCl (pH 8.0) and 300 mM NaCl, and then Triton X-100 was added to the diluted lysate to make the final concentration of 0.2%. The recombinant poly-histidine-tagged protein was purified in a working solution containing 25 mM Tris-HCl (pH 8.0), 300 mM NaCl, 1% Sarkosyl, and 0.2% Triton X-100 with the Ni resin-based columns (Bio-Rad) by HPFC (Next Generation Chromatography, Bio-Rad) and concentrated with 10k Vivaspin columns after a series of dialyses in 25 mM Tris-HCl buffer (pH 8.0).

### hLRRK1 KD modeling and docking

Because crystallographic data of the LRRK1 KD has not yet been resolved, we performed protein structure homology modeling with SWISS-MODEL [17]. BLAST searches to identify suitable templates for modeling of the hLRRK1 KD led to three highly significant matches. The detected templates of TGFβ activated kinase 1 (TAK1), constitutive triple response 1 kinase (CTR1), and mixed-lineage kinase 1 (MLK1) all belong to the PKC-like superfamily of serine/threonine protein kinases and have been co-crystallized with small inhibitors [30–33]. While CTR1 is expressed in *Arabidopsis thaliana* and plants, its mammalian homolog MAPKKK Raf plays a significant role in regulating cell growth and differentiation. TAK1 and MLK proteins expressed in mice and humans are also members of the MAPKKK family, and they play a critical role in regulating OC differentiation and bone formation [33, 34]. We chose TAK1 as a template to build a 3D structure of hLRRK1 KD using the ESyPred3D automated homology modeling program (Swiss Institute of Bioinformatics, Switzerland) because mice with disruption of TAK1 also show an osteopetrosis phenotype [33, 35]. The PDB file of the hLRRK1 KD was loaded as a receptor into Molsoft 3.7 (Molsoft, San Diego, CA), and the potential ligand binding pocket was identified. KINACore and KINASet compounds synthesized based on matches to 3D pharmacophore fingerprints generated from either published kinase active sites or from the adenosine portion of ATP from Cambridge was screened via “table docking.” Four chemicals that dock into the pocket with high scores were chosen for further *in vitro* testing.

### *In vitro* inhibition of ATP binding assay

IN04 inhibition of ATP binding to hLRRK1 KD was assessed by a pulldown assay with ATP-agarose according to the manufacturer's instructions (ATP Affpur kit III, Jena Bioscience, Jena, Germany). Briefly, 50 ng recombinant hLRRK1 KD was first incubated with various concentrations of IN04 inhibitor (3.2 nM, 16, nM, 80 nM, 0.4 μM, and 5.0 μM) or the same volume of DMSO in 500 μl of 1x binding buffer on ice for 15 minutes. After the incubation, 50 μl of the equilibrated ATP-agarose was added into the reaction and the reactions were incubated by end-over-end mixing for 1 hour at 4°C after which beads were washed 4 times in 1x washing buffer. Proteins were eluted using 2x LDS sample buffer (Invitrogen, CA), heated at 90°C for 5 minutes and analyzed with 10% NuPage Bis-tris gel by western blot with anti-6xHis antibody as described previously [1].

### *In vitro* OC formation

Primary CD11b^+^ monocytes derived from the spleen of 5-week old C57BL/6J mice were positively isolated with magnetic CD11b microbeads according to the manufacturer’s instructions (Miltenyi Biotec, Bergisch Gladbach, Germany). Briefly, 10^8^ splenocytes in 1 ml MACS buffer were incubated with 150 μl of CD11b microbeads at 4°C for 15 minutes after removing red blood cells with RBC lysis buffer. The cells were then washed with 15 ml PBS washing buffer containing 0.5% BSA and 2 mM EDTA, spun down, and re-suspended in 500 μl of washing buffer. The cell suspension was then loaded onto a MACS® Column placed in the magnetic field of a MACS Separator. The magnetically labeled CD11b^+^ monocytes were eluted and flushed out with the plunger after 3 times washing and removing from the separator. The isolated CD11b^+^ monocytes were maintained in α-MEM supplemented with 10% fetal bovine serum (FBS), penicillin (100 units/ml), streptomycin (100 μg/ml), and M-CSF (20 ng/ml) at 37°C in 5% CO_2_ for 3 days to stimulate monocyte proliferation. The cells were then induced to differentiate in a medium containing the LRRK1 inhibitor IN04. The medium was changed every 2 days. Osteoclastogenesis was evaluated by counting TRAP staining positive, multinucleated cells having at least three nuclei. Mature OC size was measured by the Lieca Application Suite X software equipped with Lieca STP6000 microscope and Image J (National Institutes of Health).

### RNA extraction and real-time PCR

RNA was extracted from primary cultures as described previously [36]. An aliquot of RNA (1 μg) was reverse-transcribed with an oligo(dT)_12–18_ primer into cDNA in a 20 μl reaction volume. The real-time PCR reaction contained 0.5 μl of template cDNA, 1x SYBR GREEN master mix (ABI), and 100 nM of specific forward and reverse primers in a 12 μl reaction volume. Primers for peptidyl prolyl isomerase A (PPIA) were used to normalize the expression data for the genes of interest. The primer sequences used for real-time PCR are listed in Table 1.

**Table 1:** Primer sequences used for real time PCR.

**Gene**	**Forward primer**	**Reverse primer**
*Ppia*	5’-CCATGGCAAATGCTGGACCA	5’-TCCTGGACCCAAAACGCTCC
*Lrrk1*	5’-GCTCAACATTGAGGCCAAGG	5’-GCCGATAGTGCTACCCACAT
*Nfatc1*	5’-ATACTTCCTGTCCTCTGGCAACA	5’-GCTTGCAGCTAGGAAGTACGTCTT
*Trap*	5’-CACTCAGCTGTCCTGGCTCAA	5’-CTGCAGGTTGTGGTCATGTCC
*Cathepsin K*	5’-GAACGAGAAAGCCCTGAAGAGA	5’-TATCGAGTGCTTGCTTCCCTTC
*Bmp6*	5’-GGAGCATCAGCACAGAGACT	5’- AAGAAGGCCACCATGAAGGG
*Cthrc1*	5'-CTACAGTTGTCCGCACCGAT	5'-TTGAATCCATCCCGACCTGG
*Wnt10b*	5'-CTGAGTAAGCGACAGCTGGG	5'-GGAGAAAGCACTCTCACGGA

### Bone resorption pit and actin ring formation assays

Slices from bovine cortical bone were placed in 48-well plates and cells were differentiated on top of the bone slices as described previously [1]. Cells on bone slices were digested with trypsin at 37°C overnight. Multinucleated cells were further removed by 5-minute sonication in 1M ammonia. Air-dried bone slices were stained with hematoxylin. The entire surface of each bone slice was examined and the total resorbed area per bone slice was quantified using ImageJ software. Resorption pits were also visualized by nano-CT at a 0.66 μM voxel dimension (VersaXRM-500, Xradia, Pleasanton, CA, USA). The pit area and pit depth on bone slices were quantified with ImageJ software and TXM Reconstructor, respectively. Cells on bone slices were also stained with Alexa Fluor 488-conjugated phalloidin for F-actin and DAPI for nuclei staining. Actin ring formation, sealing zone, and 3D OC images were visualized by Olympus Fluoview 3000 confocal microscopy.

### Nodule assay

Bone marrow stromal cells isolated from the femurs and the tibias of the mice were grown to 80% confluence. The cells were then treated with a mineralization medium containing 10 mM β-glycerophosphate, 50 μg/ml ascorbic acid, and 10% FBS for 24 days. The cells were washed, fixed, and stained with 40 mM alizarin red (pH 4.2). The mineralized area was measured as described previously [37].

### Statistical analysis

Data are presented as mean ± SEM and analyzed by Student’s t-test or two-way ANOVA as appropriate.
